# P-726. "Infectious Complications in Patients with Relapsed/Refractory Multiple Myeloma Undergoing Novel Bispecific Antibody Therapy: A Myeloma-Infectious Disease Database Initiative"

**DOI:** 10.1093/ofid/ofae631.922

**Published:** 2025-01-29

**Authors:** Silpa Jetty, Jennifer Makhoul, Fareed Khawaja, Hans C Lee, Krina Patel, Sandra Horowitz, Ella Ariza Heredia, Amy Spallone, Roy F Chemaly

**Affiliations:** University of Texas Health Science Center at Houston/MD Anderson Cancer Center, Houston, Texas; University of Texas Health Science Center at Houston/MD Anderson Cancer Center, Houston, Texas; The University of Texas MD Anderson Cancer Center, Houston, Texas; The University of Texas MD Anderson Cancer Center, Houston, Texas; MD Anderson Cancer Center, Houston, Texas; UT MD Anderson Cancer Center, Houston, Texas; The University of Texas MD Anderson Cancer Center, Houston, Texas; University of Texas MD Anderson Cancer Center, Houston, Texas; University of Texas MD Anderson Cancer Center, Houston, Texas

## Abstract

**Background:**

Novel bispecific antibody (BsAb) therapies that target both CD3 and BCMA have been approved for the treatment of relapsed/refractory multiple myeloma (R/R MM). However, this therapy has been associated with a high rate of infectious complications. Further studies are needed to determine if this is due to underlying immunosuppression from prior therapies or related to the cellular therapy. Herein, we aimed to compare the incidence, etiology, and severity of infectious complications in patients with R/R MM treated with BsAbs, chimeric antigen receptor T cell (CAR T cell) or standard of care (SOC) after six months of therapy.

Demographics and Variables

**Table 1:**
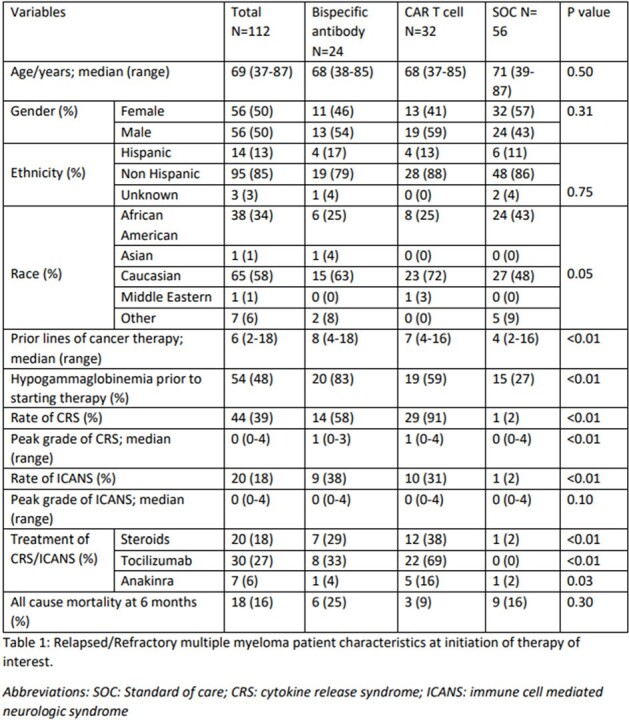
Relapsed/Refractory multiple myeloma patient characteristics at initiation of therapy of interest.

**Methods:**

We performed a retrospective study to include all R/R MM patients who underwent treatment at our center from January 2023 to June 2023. Patient demographics, cancer history and treatment related complications were reviewed. Therapy of interest included: a) BsAbs (teclistamab) or b) CAR T cell therapy (ciltacabtagene autoleucel or idecabtagene vicleucel), and c) non-cellular therapies (SOC). Our primary outcome of interest was incidence, severity, and timing of bacterial, fungal and viral related infections.

Infectious Complications

**Table 2:**
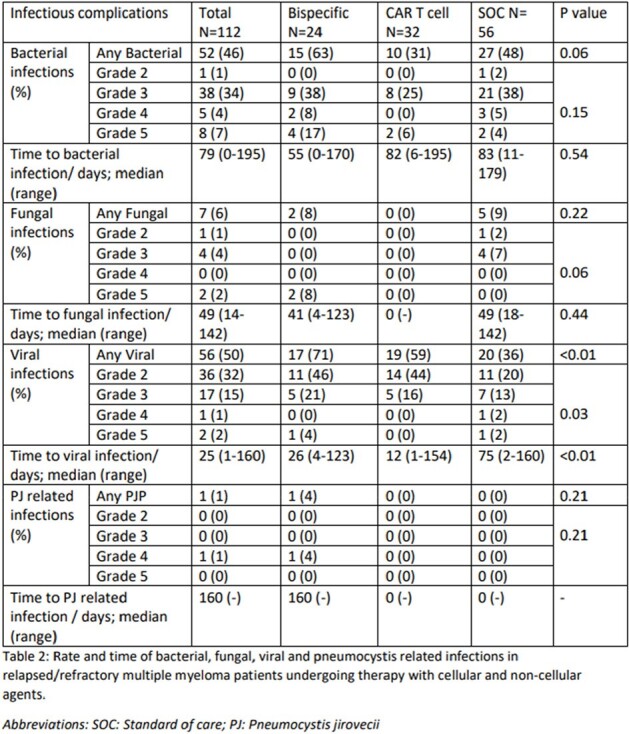
Rate and time of bacterial, fungal, viral, and pneumocystis related infections in relapsed/refractory multiple myeloma patients undergoing therapy with cellular and non-cellular agents.

**Results:**

A total of 118 patients underwent treatment for R/R MM from January 2023 to June 2023. Six patients did not receive a therapy within our inclusion criteria; therefore 112 patients were included in the preliminary analysis; 24 had received BsAb, 32 received CAR T cell and 56 underwent treatment under SOC (Table 1). Hypogammaglobinemia was higher in BsAb recipients (Table 1). Patients receiving BsAbs had a higher rate of any viral infections compared to the CAR T cell and SOC groups (71% vs 59% vs 36%, p< 0.01; table 2). Time to viral infection after therapy was sooner in the BsAbs and CAR T cell therapy group compared to SOC (Table 2 and figure 1). There was no difference regarding the occurrence of bacterial or fungal infections between the groups.

Time to First Viral Infection

**Figure 1:**
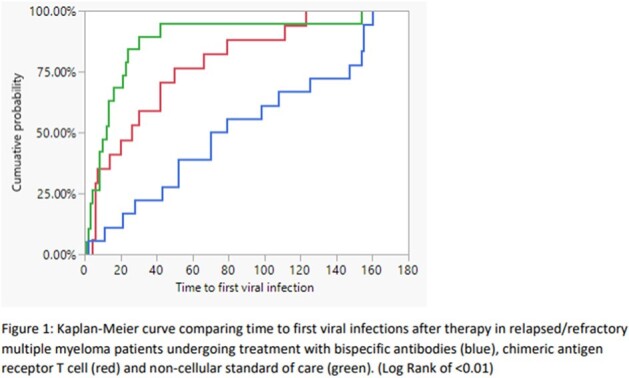
Kaplan-Meier curve comparing time to first viral infections after therapy in relapsed/refractory multiple myeloma patients undergoing treatment with bispecific antibodies (blue), chimeric antigen receptor T cell (red) and non-cellular standard of care (green). (Log Rank of < 0.01)

**Conclusion:**

Based on our preliminary analysis, R/R MM patients receiving BsAbs have a higher rate of viral infections when compared to CAR T cell therapy and standard of care treatment. Future analysis to understand independent risk factors for infection is planned once the cohort is expanded.

**Disclosures:**

**Fareed Khawaja, MBBS**, Eurofins Viracor: Grant/Research Support|Symbio: Grant/Research Support **Hans C. Lee, MD**, Abbvie: Advisor/Consultant|Allogene Therapeutics: Advisor/Consultant|Bristol Myers Squibb: Advisor/Consultant|Bristol Myers Squibb: Grant/Research Support|Genentech: Advisor/Consultant|GlaxoSmithKline: Advisor/Consultant|GlaxoSmithKline: Grant/Research Support|Janssen: Advisor/Consultant|Janssen: Grant/Research Support|Regeneneron: Advisor/Consultant|Regeneneron: Grant/Research Support|Sanofi: Advisor/Consultant|Takeda Pharmaceuticals: Advisor/Consultant|Takeda Pharmaceuticals: Grant/Research Support **Krina Patel, MD**, Abbvie: Advisor/Consultant|Arcellx: Advisor/Consultant|Astra Zeneca: Advisor/Consultant|BMS: Advisor/Consultant|BMS: Scientific committee/chair|Caribou Sciences: Advisor/Consultant|Celgene: Advisor/Consultant|Genentech: Advisor/Consultant|Janssen: Advisor/Consultant|Kite: Advisor/Consultant|Kite: Scientific committee/chair|Merck: Advisor/Consultant|Oricel: Scientific committee/chair|Pfizer: Advisor/Consultant|Regeneron: Advisor/Consultant|Sanofi: Advisor/Consultant **Roy F. Chemaly, MD/MPH**, AiCuris: Advisor/Consultant|AiCuris: Grant/Research Support|Ansun Pharmaceuticals: Advisor/Consultant|Ansun Pharmaceuticals: Grant/Research Support|Astellas: Advisor/Consultant|Eurofins-Viracor: Grant/Research Support|InflaRX: Advisor/Consultant|Janssen: Advisor/Consultant|Karius: Advisor/Consultant|Karius: Grant/Research Support|Merck/MSD: Advisor/Consultant|Merck/MSD: Grant/Research Support|Moderna: Advisor/Consultant|Oxford Immunotec: Advisor/Consultant|Oxford Immunotec: Grant/Research Support|Roche/Genentech: Advisor/Consultant|Roche/Genentech: Grant/Research Support|Shinogi: Advisor/Consultant|Takeda: Advisor/Consultant|Takeda: Grant/Research Support|Tether: Advisor/Consultant

